# Coach–athlete relationships, self‐confidence, and psychological wellbeing: The role of perceived and received coach support

**DOI:** 10.1002/ejsc.12226

**Published:** 2024-11-25

**Authors:** Adam H. Coussens, Max J. Stone, Tracy C. Donachie

**Affiliations:** ^1^ Faculty of Medical Sciences School of Biomedical Nutritional and Sports Sciences Newcastle University Newcastle Upon Tyne UK; ^2^ Faculty of Medical Sciences Newcastle University School of Psychology Newcastle Upon Tyne UK

**Keywords:** leadership, mental health, self‐efficacy, social support, team dynamics

## Abstract

Coaches are recognized as key support providers, although there is limited understanding of how coach support relates to athletes' self‐confidence and psychological wellbeing. This study examined relationships among perceived coach support, received coach support, coach–athlete relationship, self‐confidence, and psychological wellbeing. A further aim was to identify mechanisms through which coach–athlete relationship influences self‐confidence and psychological wellbeing. A total of 537 athletes (Mage = 21.83 and SD = 3.67) from a range of sports completed measures of perceived coach support, received coach support, coach–athlete relationship, self‐confidence, and psychological wellbeing. Mediation analysis revealed a significant direct effect of coach–athlete relationship on perceived coach support (*b* = 1.44 and *p* = 0.04) and received coach support on psychological wellbeing (*b* = 1.94 and *p* = <0.05). Coach–athlete relationship was associated with a significant indirect effect on psychological wellbeing via received coach support (ab = 0.82 and 95% CI [0.40 and 1.26*]) but not perceived coach support. In contrast, coach–athlete relationship was associated with a significant indirect effect on self‐confidence via perceived coach support (ab = 0.16 and 95% CI [0.10 and 0.22*]) but not received coach support. These findings demonstrate the significant role perceived coach support and received coach support plays in potentially explaining the relationship between the coach–athlete relationship with self‐confidence and psychological wellbeing. Additionally, the results highlight that different forms of social support uniquely mediate the relationship among the coach–athlete relationship, confidence, and wellbeing. These results have implications for coaching practices, as coaches can use their relationships with athletes to provide optimal support and thereby enhance the athletes' self‐confidence and wellbeing.

## Highlights

- Received coach support significantly predicted psychological wellbeing, whereas perceived coach support significantly predicted self‐confidence.
- Perceived coach support mediated the relationship between the coach–athlete relationship and athlete self‐confidence.
- Received coach support mediated the relationship between the coach–athlete relationship and athlete wellbeing.

## 1 INTRODUCTION

Athlete confidence and wellbeing can be enhanced through the support they receive and/or perceive and the relationships that they develop through playing sports (e.g., Davis & Jowett, 2014; Gencer & Öztürk, 2018). For many athletes, the coach is a key provider of social support (Freeman, 2021). The confidence and psychological wellbeing of athletes are of great concern to coaches who look to nurture positive and impactful relationships (Forlenza et al., 2018; Peng et al., 2020). Although the coach–athlete relationship and social support are associated with increased self‐confidence and wellbeing (e.g., Freeman & Rees, 2010; Simons & Bird, 2023), no studies have examined coach–athlete relationship and perceived support and received support simultaneously in the same study. Further research examining social support in relation to coach–athlete relationships is vital to help understand potential mechanisms underpinning confidence and wellbeing and distinguish nuances that may exist in support provision. The current study addresses this gap in knowledge by examining the influence of coach–athlete relationships and the mediating role of social support in enhancing confidence and wellbeing.

Self‐confidence plays a significant role in athlete performance, motivation, and overall wellbeing (Vealey & Chase, 2008). Self‐confidence is defined as a belief in one's ability to successfully execute desired actions and achieve desired outcomes (Vealey, 2001). Research has shown that higher levels of self‐confidence are associated with improved performance outcomes (e.g., Woodman & Hardy, 2003). Athletes with higher self‐confidence are likely to experience lower levels of anxiety, although feeling a sense of control and satisfaction (e.g., Craft et al., 2003). Confident athletes are also more likely to set challenging goals, persevere in the face of setbacks, and experience a greater sense of fulfillment and enjoyment in their sport (Woodman & Hardy, 2003). Coaches can play a role in this as overly critical and unsupportive coaches decreased athletes' self‐confidence (Bruner et al., 2008), whereas when athletes feel a stronger coach–athlete relationship, confidence is enhanced (Gencer & Öztürk, 2018). Thus, self‐confidence serves as a vital component of athletes' psychological wellbeing as well as contributing to overall performance.

The wellbeing of athletes is of paramount importance, as it significantly influences their overall health and performance and contributes to flourishing (e.g., Lundqvist, 2011). Psychological wellbeing refers to an individual's overall mental health and positive functioning across various domains of life (Kumar et al., 2020). Although the significance of psychological wellbeing is widely recognized for overall quality of life (Camfield & Skevington, 2008), its specific prevalence and determinants within athletic populations warrant further investigation. Some factors can pose challenges to athletes' wellbeing including elevated levels of stress, competitive anxiety, overtraining, and burnout (e.g., McLoughlin et al., 2021). Conversely, factors such as perceived ability, self‐esteem, and coping skills have shown potential in enhancing athletes' psychological wellbeing (e.g., Gould et al., 2002). One construct that is crucial in promoting wellbeing is social support.

Social support is defined as “the social resources that persons perceive to be available, or that are actually provided to them by non‐professionals, in the context of both formal support groups and informal helping relationships” (Cohen et al., 2000, p. 4). Social support incorporates both structural and functional components (Lakey, 2010). The structural component of support relates to the number and type of relationships available in one's network (e.g., the coach as a support provider; Freeman, 2021). The functional component comprises of perceived support and received support. Perceived support refers to appraisals of support availability, whereas received support refers to an assessment of the amount of support obtained usually over a given period of time (Vangelisti, 2009). Although perceived support and received support are similar in nature, they are statistically and conceptually distinct, with a shared variance of between 12% and 19% (e.g., Haber et al., 2007). Perceived support represents a dispositional interpretation of available support, whereas received support reflects objectively identifiable actions of support (Haber et al., 2007).

Both received support and perceived support have been found to positively influence psychological outcomes. For example, a meta‐analysis demonstrated that both perceived support (*r* = 0.31) and received support (*r* = 0.22) exert moderate effects on mental health outcomes in emergency support workers (Prati et al., 2010). However, other research has highlighted differences in how these two types of support relate to other variables. For example, Uchino (2004) found that perceived support is more consistently related to positive health outcomes than received support. The majority of research tends to focus on either perceived support or received support independently, although it is critical that both are examined concurrently to fully understand their individual relationships with psychological outcomes. Furthermore, only a limited amount of research on both perceived support and received support exists in sport, and studies are yet to examine these types of support in relation to coach support and the wellbeing of athletes.

Within the social support literature, there are two predominant theories to explain how social support exerts beneficial effects: (a) stress‐buffering model and (b) main effects model (Cohen et al., 2000). The stress‐buffering model suggests social support buffers the potential negative effects of stress (Cohen, 1988). There is some evidence for stress‐buffering effects in sport although this is equivocal, as when both perceived support and received support were examined simultaneously, only received support was associated with stress‐buffering effects on self‐confidence (Rees & Freeman, 2007). In comparison, the main effects model suggests direct beneficial effects of social support irrespective of stress levels (Cohen et al., 2000). In sport, main effects for both perceived support and received support were reported in relation to performance (Freeman & Rees, 2008), although this has not yet been shown for coach support specifically. The current study examines the role of perceived coach support and received coach support in reference to the main effects model, as this model has stronger evidence for the beneficial effects of social support across health (Lakey & Orehek, 2011) and sport (Freeman & Rees, 2009).

One key factor influencing coaching effectiveness is the quality of the coach–athlete relationship (Jowett, 2017). The coach–athlete relationship is defined as a social situation in which coaches' and athletes' perceived closeness (affective bond between individuals), commitment (intention in the dyad to maintain the relationship), and complementarity (interactions that underline cooperation) are interdependent (Jowett & Ntoumanis, 2004). Although the coach–athlete relationship and social support are conceptually related, they are distinct constructs. For example, both constructs are underpinned by an interaction between individuals and the perceptions of these interactions (Cohen et al., 2000). However, these constructs differ at the level at which they operate; social support encompasses perceived or received resources an individual experiences (Lakey, 2010), whereas the coach–athlete relationship pertains to an evaluation of the cognitive, emotional, and behavioral quality of a relationship (Jowett & Ntoumanis, 2004). Therefore, coach–athlete relationship could be considered a higher order construct with social support being a key behavior that maintains the quality of relationships.

Positive coach–athlete relationships are associated with more favorable psychological outcomes for athletes. Indeed, coach leadership behaviors work synergistically with coach–athlete relationship to improve developmental experience (Vella et al., 2013). There is limited empirical research examining the relationship between perceived support and coach–athlete relationship, although preliminary data suggest they are related. For example, Simons and Bird (2023) found that coach–athlete relationship constructs (closeness, commitment, and complementarity) positively correlated to different types of social support (i.e., tangible, emotional, informational, and esteem). These findings suggest that the coach–athlete relationship influences the types of support provided to athletes, although this has yet been established for received support.

Coaches are often important role models to athletes and can bolster athlete self‐confidence through guidance, encouragement, and support (Lopez de Subijana et al., 2021). Coaches play a role in developing a nurturing environment to develop positive interpersonal relationships with their athletes, which contributes to building confidence (Forlenza et al., 2018). Previous studies demonstrated that perceived support from teammates boosts athlete self‐confidence (Freeman & Rees, 2010) and both perceived and received support are crucial for self‐confidence (Rees & Freeman, 2007). Despite the importance of coaches to athletes, neither of the aforementioned studies directly examined coach support or considered other potentially influential factors such as the coach–athlete relationship.

Alongside self‐confidence, perceptions of support have also been associated with improved psychological wellbeing in college level students (Adyani et al., 2019) and athletes (Simons & Bird, 2023). Furthermore, autonomy support by a coach contributes to athletes' subjective wellbeing (Lafrenière et al., 2011). Nuance and limitations exist with this research though. For example, when perceived support and received support were considered in the same study involving student‐athletes, received support was associated with psychological wellbeing, whereas perceived support was not (Katagami & Tsuchiya, 2016). Simons and Bird (2023) examined perceptions of coach support only rather than examining both perceived and received coach support simultaneously. The equivocal nature of the relationship between perceived support and received support with athletes' psychological wellbeing warrants further investigation, specifically in relation to coach support.

Beyond establishing an association among perceived coach support, received coach support, coach–athlete relationship, self‐confidence, and psychological wellbeing, it is vital to examine the specific mechanisms through which perceived and received support operate to help advance theory and develop more effective interventions (Sarason & Sarason, 2009). Few studies have examined potential mediators underpinning coach–athlete relationships with outcomes, such as wellbeing, despite evidence suggesting they are related and may influence athlete outcomes (Simons & Bird, 2023). In particular, the positive relationship between coach–athlete relationship and athlete outcomes may be influenced by the support provided and received from the coach. Furthermore, there is currently a lack of research identifying potential mechanisms associated with both perceived support and received support in the same study or incorporating multiple psychological mechanisms concurrently (Uchino et al., 2012). Although some researchers have investigated mediating factors that explain the relationship between social support and outcomes variables (Uchino et al., 2012), there is limited research that has examined how social support acts as a mediator between social and intrapersonal factors in sport settings. Therefore, further research is needed to demonstrate how social support influences important variables in sport contexts.

### 1.1 The present study

To the best of our knowledge, no previous research has examined the role of perceived coach support, received coach support, coach–athlete relationship, self‐confidence, and psychological wellbeing in athletes. The present study aimed to (i) examine the relationships among perceived coach support, received coach support, coach–athlete relationship, self‐confidence, and psychological wellbeing and (ii) investigate the influence of perceived coach support and received coach support as potential mediators of the relationship among coach–athlete relationship, self‐confidence, and psychological wellbeing. Based on the previous research and theory, we expected a significant positive relationship among perceived coach support, received coach support, coach–athlete relationship, self‐confidence, and psychological wellbeing (hypothesis 1), and that both perceived coach support and received coach support would mediate the relationship between coach–athlete relationship and self‐confidence and the relationship between coach–athlete relationship and psychological wellbeing (hypothesis 2).

## 2 METHODS

### 2.1 Participants and procedure

GPower (G*Power, Version 3.1.9.7) was used to determine the a priori sample size required for the study. Assuming a medium effect size (*f* ^2^ = 15), an alpha probability of 0.05, a beta probability of 0.80, and three predictors, the required sample size was 77. Participants were eligible to participate if they played sport each week, interacted with their main coach at least once a week, and had maintained this relationship for at least 1 year. Following institutional ethical approval, 537 athletes (males *n* = 288, females *n* = 246, and nonbinary *n* = 3; *M*age = 21.83 and *SD* = 3.67) were recruited via opportunity sampling with participants provided with an online link to a questionnaire. All participants were provided with a participant information sheet and signed an informed consent form before completing an online questionnaire (Qualtrics, 2022, Provo) that was accessible for 4 weeks. The order in which measures were presented was randomized to counter any potential order effects. The ethnic identity of the sample consisted of 488 (90.9%) White, 25 (4.7%) multiethnicity, 11 (2.0%) Asian, 10 (1.9%) Black, two (0.4%) other non‐White, and 1 (0.2%) prefer not to say. Each participant currently worked with a coach on average 5.18 times per week (*SD* = 1.37) and had worked with their coach for an average of 3.14 years (*SD* = 3.29). Participants were sampled from a range of team (*n =* 414) and individual sports (*n* = 123), trained on average 3.54 times (*SD* = 2.37) per week, competed 1.40 (*SD* = 0.81) times per week, and had played their sport for 15.81 years (*SD* = 6.14). The participants' current competitive level ranged from the university/college level (*n* = 315), county/semiprofessional (*n* = 127), professional (*n* = 25), national (*n* = 48), and international (*n* = 22). See Table 1 for full demographic information.

**TABLE 1:** Descriptive statistics, bivariate correlations, and internal reliability of variables.

Variable	Mean	SD	PCS	RCS	SC	C‐A	PWB
PCS	3.58	0.79	(0.94)				
RCS	2.71	0.90	0.59*	(0.96)			
SC	3.15	0.66	0.59*	0.40*	(0.90)		
C‐A	5.51	1.14	0.78*	0.53*	0.57*	(0.94)	
PWB	47.87	8.50	0.32*	0.33*	0.39*	0.30*	(0.90)

*Note*: * Significant to *p* < 0.05. Values in parentheses represent Cronbach's alpha for the respective variables. PCS, perceived coach support; RCS, received coach support; SC, self‐confidence; C‐A, coach–athlete relationship; and PWB, psychological wellbeing.

### 2.2 Measures

#### 2.2.1 Perceived coach support

Perceived coach support was assessed using the 16‐item Perceived Available Support Questionnaire (PASS‐Q; Freeman et al., 2011). Each of the question items were preceded by the question stem “If needed to what extent would your main coach….” Sample items included “Provide you with comfort and security?” and “Boost your sense of competency?” Participants responded on a five‐point Likert scale from 1 (*not at all*) to five (*extremely so*). Freeman et al. (2011) provided evidence to support the internal and test–retest concurrent validity and reliability of the measure. Internal reliability in the current study was very good (*α* = 0.94).

#### 2.2.2 Received coach support

Perceptions of received coach support were assessed using the 22‐item Athletes' Received Support Questionnaire (ARSQ; Freeman et al., 2014). Each of the question items were preceded by the question stem “In the last 4 weeks, how often has your main coach….” Sample items included “Cheer You Up?” and “Listen to you?” Participants responded on a five‐point Likert scale from 1 (*not at all*) to 5 (*seven or more times*). Freeman et al. (2014) provided evidence to support the content validity and reliability of the measure. Internal reliability in the current study was very good (*α* = 0.96).

#### 2.2.3 Self‐confidence

Self‐confidence was assessed using the five item Revised Competitive State Anxiety Inventory 2 (CSAI‐2R; Cox et al., 2003) such as that of the previous social support research (Freeman & Rees, 2010). Participants responded to statements regarding perceptions of self‐confidence about an upcoming match/game in the presence of their main coach. Each of the question items were preceded by the question stem “In the presence of my coach.?” and sample items which included “I would be confident I could meet the challenge?” and “I would be confident about performing well?” Participants responded on a four‐point Likert scale ranging from 1 (*not at all*) to 4 (*very much so*). Evidence to support the factorial validity was provided by Cox et al. (2003). Internal reliability in the current study was very good (*α* = 0.89).

#### 2.2.4 Coach–athlete relationship

Perceptions of athletes' relationship with their coach was assessed using the 11 item Coach–Athlete Relationship Questionnaire (CART‐Q; Jowett & Ntoumanis, 2004), consisting of three subscales including closeness, commitment, and complementarity. As suggested by Lafrenière et al. (2011), these subscales are combined to show an overall measure of coach–athlete relationship. Participants were provided with statements regarding the nature of their relationship with their coach and asked to respond to these on a seven‐point Likert scale ranging from 1 (*strongly disagree*) to 7 (*strongly agree*). Sample items included “I feel close to my coach” and “I feel committed to my coach.” Evidence for content validity and internal reliability was provided by Jowett and Ntoumanis (2004). Internal reliability of the CART‐Q in the current study was very good (*α* = 0.94).

#### 2.2.5 Psychological wellbeing

Functional and affective components of overall psychological wellbeing were assessed using the 14‐item Warwick Edinburgh Mental Wellbeing Scale (WEMWBS; Tennant et al., 2007). Each of the question items were preceded by the question stem “Please select the response that best describes your experience over the last 4 weeks…?” and sample items included “I've been feeling relaxed” and “I've been dealing with problems well?” Participants responded on a five‐point Likert scale ranging from 1 (*none of the time*) to 5 (*all of the time*). Evidence for the validity and reliability of the measure was provided by (Tennant et al., 2007). Internal reliability of the WEMWBS in the current study was very good (*α* = 0.90).

### 2.3 Data analyses

All analyses were completed using SPSS (version 28.0; IBM Corp.). Data were first checked for missing values, assumptions of normality, and outliers. If participants had missing values, their data were removed from the study. Mean or total scores were provided for each measure, rather than providing individual subscales, consistent with previous research (e.g., Rees et al., 2012). Assumptions of normality were checked using visual inspections of scatterplots and Q–Q plots and computing kurtosis and skewness scores for each variable (with a cut‐off value set at 2.0 for skewness and 7.0 for kurtosis as the *n* number exceeds 300; Kim, 2013). Using these methods, only the CART‐Q was considered abnormally distributed. Outliers were identified via the interquartile range method (using a multiplier of three). Using this method, one outlier was identified in the CART‐Q which was winsorized to the next acceptable value.

Following screening of data, descriptive statistics, reliability statistics, and bivariate correlations were calculated for all variables. We conducted two mediation analyses via Hayes' (2013) PROCESS macro (model number 4). In the first mediation model, the *X* variable was coach–athlete relationship, the *Y* variable was psychological wellbeing, and the mediators were perceived coach support and received coach support. In the second mediation model, the same *X* and mediator variables were used but the *Y* variable was self‐confidence. In both mediation models, mediators ran parallel to one another. Mediation analyses permit the reporting of data from regression analyses including *r* ^*2,*^ *a, b*, direct effects, *t,* and *a* × *b* indirect effect values. All mediation models were bootstrapped using 5000 samples. Mediation models were considered significant if confidence intervals for the indirect effects did not include zero.

## 3 RESULTS

### 3.1 Correlation analyses

The mean scores for the primary variables of interest (perceived coach support, received coach support, coach–athlete relationship, self‐confidence, and psychological wellbeing) are presented in Table 2. Bivariate correlation analyses revealed a positive significant relationship between all variables as shown in Table 2. Using Cohen's recommendation (1988), self‐confidence demonstrated a significant, positive, and strong relationship with perceived coach support (*r* = 0.59 and *p* < 0.001), coach–athlete relationship (*r* = 0.57 and *p* < 0.001), and moderate relationship with received coach support (*r* = 0.40 and *p* < 0.001). Psychological wellbeing demonstrated a significant, positive, and moderate relationship with perceived coach support (*r* = 0.32 and *p* < 0.001), received coach support (*r* = 0.33 and *p* < 0.001), self‐confidence (*r* = 0.39 and *p* < 0.001), and coach–athlete relationship (*r* = 0.30 and *p* < 0.001). Perceived coach support and received coach support demonstrated a significant, positive, and strong relationship with one another (*r* = 0.59 and *p* < 0.001).

**TABLE 2:** Mediation results for coach–athlete relationship predicting psychological wellbeing and self‐confidence, with perceived coach support and received coach support as mediators.

Mediators	r^2^	a	b	Direct effect c’	t	Indirect Effect a × b (95% CI)
*PWB*	0.14*					
PCS		0.54	1.44			0.78 (−0.10, 1.71)
RCS		0.42	1.94			0.82 (0.40, 1.26)*
CA‐R				0.62	1.23	
*SC*	0.39*					
PCS		0.54	0.30			0.16 (0.10, 0.22)*
RCS		0.42	0.03			0.01 (−0.01, 0.04)
CA‐R				0.16	4.98	

*Note*: * Significant effects to *p* < 0.05. PCS, perceived coach support; RCS, received coach support; SC, self‐confidence; C‐A, coach–athlete relationship; and PWB, psychological wellbeing.

### 3.2 Mediation analyses

Results for the mediation analyses are presented in Table 2. In the first model, coach–athlete relationship, perceived coach support, and received coach support collectively accounted for 14% of the variance of psychological wellbeing. Coach–athlete relationship was associated with a significant indirect effect (i.e., mediation) on psychological wellbeing via received coach support (ab = 0.82 and 95% CI [0.40 and 1.26*]), but not perceived coach support (ab = 0.78 and 95% CI [−0.10 and 1.71]). In the second model, coach–athlete relationship, perceived coach support, and received coach support collectively accounted for 39% of the variance of self‐confidence. Coach–athlete relationship was associated with a significant indirect effect on self‐confidence via perceived coach support (ab = 0.16 and 95% CI [0.10 and 0.22*]) but not received coach support (ab = 0.01 and 95% CI [−0.01 and 0.04]).

## 4 DISCUSSION

The first aim of this study was to examine the relationship among perceived coach support, received coach support, coach–athlete relationship, self‐confidence, and psychological wellbeing. In support of hypothesis 1, correlational analyses found significant positive relationships between all variables. The second aim was to investigate perceived coach support and received coach support as potential mediators influencing the relationship between coach–athlete relationship with psychological wellbeing and self‐confidence. Hypothesis 2 was partially supported as the coach–athlete relationship is associated with a significant indirect effect on psychological wellbeing via received coach support but not perceived coach support. Conversely, the coach–athlete relationship is associated with a significant indirect effect on self‐confidence via perceived coach support but not received coach support.

Bivariate correlations showed that both perceived coach support and received coach support were significantly correlated with psychological wellbeing; however, when considered simultaneously in the mediation analysis, only received coach support emerged as a significant predictor of psychological wellbeing. This finding highlights the importance of examining multiple types of support simultaneously to identify optimal practice recommendations for coaches in sport settings. Received support was a significant predictor of psychological wellbeing in the current study, which aligns with evidence from social psychology (Dadvand et al., 2016), and the current study extends this understanding specifically to athletes, suggesting that received coach support influences the relationship between coach–athlete relationship and psychological wellbeing.

Contrary to previous research (e.g., Simons & Bird, 2023), the current study found that perceived coach support did not predict psychological wellbeing. However, these findings may be explained by the analytical approach adopted by the current study. Specifically, the current study simultaneously considered both perceived coach support and received coach support, whereas previous research has mainly examined perceived or received social support in isolation (e.g., Simons & Bird, 2023). Consequently, unlike previous research, we were able to control for, and observe, the unique contributions of perceived and received social support in relation to our outcome variables. This suggests that received social support and perceived social support have distinct relationships with different outcome variables in sport contexts. It has been suggested that received support may be context specific as unwarranted support can be perceived as unhelpful or a threat to independence (Bolger & Amarel, 2007). A potential explanation for why only received coach support predicted psychological wellbeing could be unique to coach support. For example, coaches may be key providers of support for athletes in sporting contexts (e.g., Freeman, 2021) and receiving more tangible forms of support from these individuals may be critical to athlete wellbeing rather than perceiving coach support to be available. Furthermore, when received support is deemed particularly needed, its relationship with mental health is stronger (Melrose et al., 2015), suggesting that in the current sample, receiving coach support was deemed necessary for increased psychological wellbeing.

The findings of the current study extend the work of Simons and Bird (2023) by demonstrating that coach–athlete relationship is also associated with overall psychological wellbeing beyond sport‐related wellbeing. When included in the mediation analysis, coach–athlete relationship indirectly predicted psychological wellbeing via perceived coach support and received coach support. This finding suggests that the way coach–athlete relationship influences outcomes is through social support. In comparison, Simons and Bird (2023) found that coach–athlete relationship positively predicted psychological wellbeing in athletes. The current findings provide a more nuanced understanding of these relationships whereby received coach support influences the relationship between coach–athlete relationship and psychological wellbeing. Further research examining both perceived support and received support alongside coach–athlete relationship as potential predictors of psychological wellbeing is required. The current results suggest that it is not the relationship that is key in facilitating wellbeing in athletes but rather the extent to which athletes feel supported. These findings emphasize the crucial role that a coach plays in being a support provider to athletes and the wider beneficial effects on their wellbeing.

The results of this study underscore the pivotal role that coaches play in shaping athletes' self‐confidence, echoing the findings of Freeman et al. (2011). Notably, perceived coach support emerged as a positive predictor of self‐confidence, with higher levels of perceived coach support corresponding with heightened self‐confidence. Moreover, the study reinforces the importance of coach–athlete relationship in regard to self‐confidence. A stronger connection between the coach and athlete is linked to enhanced self‐confidence, consistent with Gencer and Öztürk (2018). In contrast to previous research (e.g., Freeman et al., 2014), received support did not predict self‐confidence, although our findings may be specific to types of support provided by coaches. The current findings are supported by previous research that has shown perceived support to be strongly related to similar variables such as self‐esteem (e.g., Lu et al., 2023). Previous studies within sport psychology have failed to account fully for how social support relates to self‐confidence, specifically in relation to the role of coach support. Our research suggests that perceptions of coach support are more important than the support actually received in influencing self‐confidence. Although not tested in the current study, received coach support might aid self‐confidence via stress‐buffering effects (e.g., Rees & Freeman, 2007).

Sarason and Sarason (2009) requested more research to explore the mechanisms of how social support works, with a particular need to test multiple psychological mechanisms simultaneously (Uchino et al., 2012). In line with these previous recommendations, social support was examined as a potential mechanism underpinning key wellbeing and performance indicators. The current study found that perceived coach support and received coach support may exert contrasting effects. Specifically, coach–athlete relationship was associated with a significant indirect effect on psychological wellbeing via received coach support but not perceived coach support. Therefore, when athletes feel they have a strong coach–athlete relationship and receive support from their coach, psychological wellbeing is enhanced. In contrast, coach–athlete relationship was associated with a significant indirect effect on self‐confidence via perceived coach support but not received coach support. Therefore, when athletes feel that they have a strong coach–athlete relationship and perceive coach support to be available, self‐confidence is enhanced. Previous research has identified distinct mechanisms explaining how perceived support and received support may relate to health outcomes differently (Uchino, 2009). This aligns with the current findings, which show opposing indirect effects of perceived coach support and received coach support on well‐being and self‐confidence.

The current study highlights the importance of social factors in sport, particularly the role of the coach. Although previous research has identified a relationship among social support with coach–athlete relationship (Felton & Jowett, 2013), self‐confidence (Freeman et al., 2011), and positive wellbeing (Simons & Bird, 2023), the current study is the first to explore the relationship between these variables together. Furthermore, the current research examined the role of both perceived coach support and received coach support concurrently in relation to confidence and wellbeing. Studies mainly focus on one social support construct and therefore, are unable to detect the nuance effects of both perceived support and received support on outcome variables or identify potential mechanisms underpinning relationships between variables. The current study addresses recommendations for research to examine both support constructs (Freeman, 2021). Simons and Bird (2023) found an association between coach–athlete relationship and psychological wellbeing with perceived coach support but did not measure the potential impact of received coach support. The current research significantly enhances our comprehension of how social support positively influences wellbeing and confidence by identifying mediating effects, while also distinguishing how perceived coach support and received coach support function separately (DeFreese & Smith, 2013).

This study has important implications for how coaches can best offer support to their athletes and why such support is needed. Our findings highlight the positive influence perceived coach support and received coach support has in relation to a strong coach–athlete relationship, with enhanced self‐confidence and psychological wellbeing. However, the effectiveness of social support‐based interventions has been mixed (e.g., Hogan et al., 2002). For example, one study found an intervention that centerd around an expert providing support to golfers led to feelings of enhanced received support of all three participants although performance only improved for one participant (Freeman et al., 2009). Despite the challenges of developing effective support interventions, the current findings may provide an evidence‐based approach to effective coach support. We will discuss an approach to increase perceptions of available coach support and a recommendation for providing coach support.

In practical terms, enhancing perceptions of coach support along with developing a positive coach–athlete relationship could lead to improved self‐confidence. Increasing perceptions of coach support may be considered relatively challenging to implement, as perceptions are based on generic evaluations of support providers and are considered relatively stable (Lakey, 2010). One potential strategy to improve perceptions of available support, and therefore, enhance self‐confidence, could be for coaches to have an open‐door policy to allow their athletes to speak to them if needed. This approach encourages coaches to cultivate a meaningful relationship with their athletes and shows commitment to supporting their needs. Additionally, by improving the receipt of actual support alongside developing a positive coach–athlete relationship could help enhance psychological wellbeing. Coaches could cultivate greater awareness of their role as a support provider to advocate psychological well‐being and consciously support their athletes in times of need. Understanding individuals' support needs and how and when this support can be provided may take time but would be worthwhile to nurture a positive coach–athlete relationship and psychological wellbeing. Furthermore, educating athletes as to the importance of perceived coach support and received coach support, and encouraging them to seek coach support, may have benefits for enhanced confidence and wellbeing.

Against the backdrop of the novelty of this study, there are some limitations. The current study is cross‐sectional in nature and examines only a single time point of athletes' ratings of their coach. Further research might consider a longitudinal approach to monitor coach support over a competitive season, as this could highlight any potential effects of performance results and whether these influence evaluations of coach support, coach–athlete relationships, and psychological wellbeing. Therefore, tracking coach support for longer would provide a better understanding of the stable or transient nature of coach support. Moreover, although mediation analyses revealed perceived support and received support as potential mechanisms underpinning coach–athlete relationship with self‐confidence and psychological wellbeing, this approach is unable to establish true cause and effect. Future studies might consider adopting an experimental design such as manipulating coach support and measuring subsequent effect on self‐confidence in relation to a performance task and psychological wellbeing.

To conclude, the findings from the current study demonstrate the distinct roles that perceived coach support and received coach support play in relation to the coach–athlete relationship, self‐confidence, and psychological wellbeing. When analyzed concurrently, received coach support, but not perceived coach support, predicted psychological wellbeing, whereas only perceived coach support and the coach–athlete relationship were significant predictors of self‐confidence, whereas received coach support was not. This suggests that the mechanisms in which perceived coach support and received coach support relate to wellbeing and self‐confidence operate differently. Such that, the coach–athlete relationship is associated with a significant indirect effect on self‐confidence via perceived coach support. Whereas, the coach–athlete relationship is associated with a significant indirect effect on psychological wellbeing via received coach support. Based on the results in this study, we have suggested practical recommendations for coaches to become more effective support providers by considering athletes' perceptions of support and the support received, which has implications for improving athletes' self‐confidence and psychological well‐being.

## CONFLICT OF INTEREST STATEMENT

The authors declare that they have no conflicts of interest.

## ETHICAL STATEMENT

The study was approved by the Newcastle University Ethical Committee (Ref: 13,142/2018).

## Data Availability

Any raw data are available upon request by contacting the corresponding author.
